# The retinoblastoma protein contributes to maintaining the stability of HPV E7 in cervical cancer cells

**DOI:** 10.1128/jvi.02203-24

**Published:** 2025-03-25

**Authors:** Ifeoluwa Gbala, Nezka Kavcic, Lawrence Banks

**Affiliations:** 1 Tumour Virology Laboratory, International Centre for Genetic Engineering and Biotechnology 18470 , Trieste, Italy; College of Agriculture & Life Sciences, University of Arizona, Tucson, Arizona, USA

**Keywords:** HPV, E7, cervical cancer, pRB, half-life, proteasome degradation

## Abstract

High-risk human papillomaviruses (HR HPV)-16 and -18, and other closely related subtypes, are associated with at least 90% of human cervical cancers. Cervical cancers and derived cell lines continuously express high levels of the HPV oncoprotein E7, known to degrade the tumor suppressor retinoblastoma protein (pRB). This E7–pRB interaction is important for the maintenance and progression of malignancy. In the case of HPV E6, substrate recognition has been reported to play an important role in stabilizing the viral oncoprotein; however, such regulation of E7 has so far not been investigated. Using biochemical, immunostaining, and clonogenic assays, we describe an intriguing role for pRB in the stabilization of E7 oncoprotein in HPV-positive cervical-cancer-derived cell lines. The knockdown of pRB expression by RNA interference results in a significant decrease in the levels of E7 protein in CaSki, SiHa, HeLa, and C-4 I cells. We show that pRB knockdown regulates HPV E7 at the transcription and protein levels, and significantly reduces the half-life of E7 protein by at least twofold in SiHa and HeLa cells. We also demonstrate that the destabilization of E7 caused by pRB knockdown results in significant inhibition of cell proliferation and colony formation of HPV-16 and -18 E7-positive SiHa and HeLa cells. Furthermore, the expression of wild-type pRB in pRB-depleted cells significantly restored E7 levels. Therefore, we propose that pRB, in addition to being a degradation target for HPV E7, is crucial for its stabilization.

**IMPORTANCE:**

The human papillomavirus (HPV) viral proteins E6 and E7 cooperatively contribute to tumorigenesis by disrupting cellular targets. These oncoproteins are degraded via the proteasome pathway; however, they are continuously expressed in cervical cancer cell lines. The retinoblastoma protein, pRB, is a degradation target of high-risk (HR) HPV E7 oncoprotein. Several studies have shown that the binding of E7 to pRB is important for its E7-mediated inactivation and demonstrated how pRB protein levels respond to the presence and absence of E7. However, the modulatory role of pRB on E7 protein levels has so far not been reported. Here, we report a novel regulatory relationship between E7 and pRB. We found that the continuous expression of pRB is critical for E7 stabilization. We demonstrate that this pRB-related E7 destabilization occurs in part through enhanced protein turnover. Thus, our findings provide new insights into the importance of the E7–pRB interaction in driving tumorigenesis.

## INTRODUCTION

Human papillomavirus (HPV) infection stands as a major global health concern, with persistent infections by high-risk (HR) HPV types, such as HPV-16 and HPV-18, being strongly associated with the development of various cancers, including cervical cancer (1). The intricate interplay between viral oncogenes and host cell regulatory pathways plays a pivotal role in driving tumorigenesis. Among the viral proteins implicated in HPV-induced carcinogenesis, the E7 oncoprotein plays a pivotal role by perturbing crucial cellular processes through its interactions with host cell proteins (2–4). One such critical interaction is that between HPV E7 and the retinoblastoma protein (pRB), a key regulator of cell cycle progression and a tumor suppressor (5–8).

Studies have shown that HPV-positive cervical cancer tumors continually express E6 and E7 oncoproteins, suggesting a continuing role in the development and maintenance of malignancy (9–12). These oncoproteins have recently been reported to interact directly with each other, and the crucial roles of this E6-E7 complex in promoting tumorigenesis remain to be unraveled (13). In addition, the continued expression of these oncoproteins is required for the proliferation and progression of HPV-positive cancer cells *in vitro* and *in vivo* (4, 14–16); hence, their inhibition results in suppression of cell growth (reviewed in references 17–20).

Although HPV E7 is degraded by the Cullin-1-containing ubiquitin ligase complex via the 26S proteasome pathway in host cells (21), high E7 levels are still maintained in HPV-positive cells, indicating that E7 can evade degradation through various mechanisms to attain stability (reviewed in reference 22). Previously, HPV E7 was shown to be stabilized by USP11 (23) and E6-Associated Protein (E6AP) (24) in a proteasome-dependent manner in cervical cancer cell lines. Similarly, the membrane-associated ubiquitin ligase MARCHF8 was recently reported to stabilize E7 by degrading the ligases Cullin-1 (CUL-1) and UBE2L3 in head and neck cancer (25). These studies also demonstrated that increased E7 stability enhances its oncogenicity. Therefore, it is important to understand the molecular mechanisms that govern the cellular stability of the E7 oncoprotein and the implication of these mechanisms in the management of HPV-positive cancers.

pRB is a crucial node whose dysregulation underlies the development and progression of various human cancers. In the context of HPV infection, pRB assumes a unique role, as the E7 oncoprotein intricately manipulates its function to promote viral replication and cellular transformation (26). E7 targets pRB in two distinct ways—degradation via the ubiquitin-mediated pathway (27–29) and by deregulation of the pRB-E2F1 transcriptional repression complex, which disrupts pRB’s ability to restrain cell cycle progression (5, 30–32). It has, however, been reported that E7 not only contributes to the deregulation of pRB-dependent E2F1 activation but also activates E2F1 independently of pRB (33). Structurally, E7 has been shown to depend far more on its interaction with the LXCXE-binding groove of pRB than pRB-binding proteins that are vital for cell cycle arrest or transcriptional repression (34). The disruption of this structural relationship was shown to restore pRB’s function as a cell cycle regulator while being resistant to degradation by E7 (34, 35); however, the regulatory impact of this disruption on E7 has so far not been investigated. Despite substantial progress in understanding the molecular mechanisms underlying pRB dysregulation in HPV-positive cervical cancer, several key questions remain unanswered. These questions include elucidating the molecular events governing the interaction between E7 and pRB. For instance, a recent study reported that E7-mediated proteasomal degradation of pRB requires another protease, calpain, which cleaves pRB after Lys 810 (36). So, progressive investigation into the regulation of E7–pRB interaction remains crucial to a better understanding of the molecular mechanisms underlying transformation by HPV E7.

In addition, in the case of HPV E6, previous studies have shown that substrate recognition, and in particular that with the E6AP ubiquitin ligase, contributes toward the stabilization of E6 (37). However, to date, no similar studies have investigated whether E7 substrates can also contribute toward the stabilization of the viral protein. In this study, we describe an intriguing interplay between pRB and E7. Here, we show that the continued expression of pRB is required for the stability of HPV E7. We also show that the E7 destabilization caused by pRB knockdown is sufficient to disrupt its oncogenic potential by inhibiting colony formation of HPV-16/18-E7-expressing SiHa and HeLa cells.

## MATERIALS AND METHODS

### Cell culture and reagents

Non-cancerous human keratinocytes, HaCaT cells, and HPV-positive cells expressing HPV-16 or -18 E7 protein (SiHa, CaSki, HeLa, and C-4 I cells) were obtained from the American Type Culture Collection (ATCC) and maintained in Dulbecco’s modified Eagle’s medium (DMEM) (Gibco, no. 31885-023), supplemented with 10% fetal bovine serum (FBS) (Gibco, no. 10270-106), penicillin-streptomycin (100 U mL^−1^), and glutamine (300 µg mL^−1^) (Gibco, no. 10378-016). Cells were grown at 37°C in a humidified air incubator containing 10% CO_2_.

### Antibodies and inhibitors

The primary antibodies used were mouse anti-RB1 (pRB) monoclonal antibody (BD Pharmingen, no. 554136), mouse monoclonal anti-16 E7 (NM2) (Santa Cruz Biotechnology [SCBT], no. sc65711), mouse monoclonal anti-18 E7 (SCBT, no. sc365035), mouse monoclonal anti-GAPDH (6C5) (SCBT, no. sc-32233), mouse monoclonal anti-α-tubulin (Cell Signalling Technology, no. T5168), mouse monoclonal anti-p84 (1:3,000; Abcam, no. ab487), mouse monoclonal anti-β-galactosidase (β-Gal) (Promega, no. Z378B), followed by horseradish peroxidase (HRP)-conjugated anti-mouse secondary antibody (Jackson ImmunoResearch Laboratories, no. 115-035-071 7).

For proteasome inhibition, CBZ (MG132 Z-Leu-Leu-Leu-al; Sigma-Aldrich, no. C2211) was used, while cycloheximide (Sigma-Aldrich, no. C4859) was used to inhibit protein synthesis.

### Plasmids

Two lentiviral vectors carrying short hairpin RNA (shRNA) targeting human RB1 (pRB) were used in this study. pLKO-RB1-shRNA19 is a third-generation lentiviral plasmid containing 42 bp of shRNA against pRB and was a kind gift from Todd Waldman (Addgene plasmid #25640). The second plasmid, TLCV2-RB1, is an all-in-one doxycycline inducible LentiCRISPR system. The addition of doxycycline drives the constitutive expression of an sgRNA targeting pRB. TLCV2-RB1 was a kind gift from Adam Karpf (Addgene plasmid #87836). The control shRNA plasmids used in this study, pLKO.1 GFP shRNA and pLKO.1 Scramble shRNA, were kind gifts from David Sabatini (Addgene plasmid #30323; Addgene plasmid #1864). Giannino Del Sal kindly provided the packaging and envelope plasmids.

The HA-tagged pRB expression construct used was a kind gift provided by James DeCaprio. A mutant of pRB with disruptions at the LXCXE binding cleft, previously described as defective in HPV E7 binding (34, 35), was generated by site-directed mutagenesis. The HA-tagged pRB Y756F-N757A was generated using the QuikChange site-directed mutagenesis system (Stratagene) according to the manufacturer’s instructions with the following primers: forward primer 5′-AGTCTCTGCATGAAGACCGAGGCAAAGAATACTATAATAGAATCATACTCCTCTTC-3′, reverse primer 5′-GAAGAGGAGTATGATTCTATTATAGTATTCTTTGCCTCGGTCTTCATGCAGAGACT-3′.

### siRNA transfection

CaSki (1.2 × 10^5^ cells), SiHa (1.2 × 10^5^ cells), HeLa (0.9 × 10^5^ cells), or C-4 I (0.9 × 10^5^ cells) were seeded into 6 cm^2^ plates and grown for 24 h in DMEM supplemented with penicillin, glutamine, and FBS. Then, the cells, being 30%–40% confluent, were transfected with siRNAs targeting pRB (ON-TARGET*plus* SMARTpool, Dharmacon), HPV-18 E6/E7 (Dharmacon; sense strand 5′-CAUUUACCAGCCCGACGAG dT dT-3′), HPV-16 E6/E7 (Eurofins; 5′-CAACUGAUCUCUACUGUUA-3′, 5′-CCGGACAGAGCCCAUUACA-3′, 5′-CACCUACAUUGCAUGAAUA-3′), or a non-targeting Scramble siRNA (siSTABLE Dharmacon; 5′-UAGCGACUAAACACAUCAA-3′), at a final concentration of 20 µM using Lipofectamine RNAiMax (Life Sciences Technologies, no. 13778-150). Cells were maintained in DMEM supplemented with FBS only. After 72 h, cells were harvested and directly analyzed by Western blotting or processed further for fractionation or cycloheximide chase assays. The SMARTpool sequences of the pRB-targeting siRNA used are 5′-GAACAGGAGUGCACGGAUA-3′, 5′-GGUUCAACUACGCGUGUAA-3′, 5′-CAUUAAUGGUUCACCUCGA-3′, and 5′-CAACCCAGCAGUUCGAUAU-3′.

### Lentivirus production and generation of stable cell lines

To generate lentiviral particles, packaging plasmid psPAX2, envelope plasmid pCMV-VSV-G, and either of the plasmids carrying shRNA targeting RB1 were co-transfected into HEK 293T cells using 3 µL of 1 mg/mL polyethylenimine per microgram of DNA. After 48 h of transfection, virus-containing conditioned media were collected and filtered through a 0.45 µm filter. Then, about 70% confluent recipient cells—CaSki, SiHa, HeLa, C-4 I, and HaCaT, seeded into 6 cm^2^ plates, were transfected with lentivirus at a ratio of 1:3 of viral stock to complete DMEM medium supplemented with 8 µg/mL of polybrene. Finally, 2 µg/mL of puromycin was used for the selection of infected cells from 24 h after infection for all cell lines except for HaCaT cells (after 48 h). The selection continued for another 2 wk, and the generated stable cell lines were then maintained in 1 µg/mL of puromycin. Transfection and knockdown efficiencies were evaluated by fluorescence microscopy, qRT-PCR, and Western blotting. Subsequent assays were carried out using the generated stable cell lines.

### Real-time quantitative PCR (qRT-PCR)

Total RNA was extracted from lentivirus-transfected CaSki, SiHa, HeLa, C-4 I, and HaCaT cells according to the TRIzol Reagent (Sigma, St. Louis, MO, USA) protocol. The concentration and quality of the total RNA were determined by Nanodrop One Microvolume UV-Vis Spectrophotometers (Thermo Fisher Scientific, Waltham, MA, USA). Then, reverse transcription was performed to obtain cDNA by using the QuantiTect Reverse Transcription Kit (QIAGEN, Aarhus, Denmark). The obtained cDNA was used for qPCR using PowerUp SYBR Green PCR Master Mix (Thermo Fisher Scientific). The mRNA levels of target genes were normalized to GAPDH, and fold change in gene expression was calculated using Livak’s 2^−△△Ct^ method. The primer sequences (5′–3′) for all target genes are as follows: HPV-16 E7 forward primer 5′-ACAAGCAGAACCGGACAGAG-3′, reverse primer 5′-CTGAGAACAGATGGGGCACA-3′; HPV-18 E7 forward primer 5′-TGCATGGACCTAAGGCAACA-3′, reverse primer 5′-CTCGTCGGGCTGGTAAATGT-3′; RB1 forward primer 5′-AGGTCTGCCAACACCAACAA-3′, reverse primer 5′-GCATTCGTGTTCGAGTAGAAGTC-3′.

### Western blotting

Total cell extracts were obtained by lysing the cells directly in 2× SDS-PAGE sample buffer, denatured at 95°C for 10 min, separated by SDS-PAGE, and then blotted on a 0.22 µm nitrocellulose membrane. Membranes were blocked in 5% non-fat dry milk in TBS-T (20 mM Tris-HCl, pH 7.5, 150 mM NaCl, 0.1% Tween 20) for 1 h and probed with the appropriate primary and secondary antibodies. The blots were then developed using the ECL Western blotting detection reagent (GE Healthcare, Amersham, no. RPN2106) according to the manufacturer’s instructions.

### Proteasomal inhibition

For the transient assays, CaSki, SiHa, and HeLa cells were seeded and transfected with the respective siRNAs, as described above. After 72 h, the cells were treated with 20 µM CBZ and further incubated for 6 h, before being harvested and analyzed by Western blotting.

Lentivirus-transfected SiHa and HeLa cells were seeded at 1.2 × 10^5^ cells/well and 0.9 × 10^5^ cells/well, respectively, into 6-well plates. After 48 h, the cells were treated with 20 µM CBZ and further incubated for 6 h before being harvested and analyzed by Western blotting.

### Cycloheximide chase

Following the 72 h incubation of CaSki and SiHa cells transfected with siRNA targeting pRB, HPV-16 E7, or the control Scramble siRNA, the cells were treated with 50 µg/mL cycloheximide and further incubated for 0, 20, 40, or 60 min. The cells were trypsinized at each time point and processed for cell fractionation. The fractions were then analyzed by Western blotting.

For the lentivirus-transfected SiHa cells, the cells were treated with 50 µg/mL cycloheximide and further incubated for 0, 20, 40, or 60 min. The cells were harvested at each time point and analyzed by Western blotting.

### Cell fractionation

Seventy-two hours after siRNA transfection, CaSki and SiHa cells were trypsinized and collected by centrifugation at 500 × *g* for 5 min at 4°C. The cell pellets were washed once with 1× phosphate-buffered saline (PBS) and processed into cytoplasmic and nuclear fractions, using the NE-PER Nuclear and Cytoplasmic Extraction Reagent Kit (Thermo Scientific, no. 78833), according to the manufacturer’s instructions. After obtaining the fractions, 6× SDS-PAGE sample buffer was added, and they were analyzed by Western blotting.

### EdU cell proliferation assay

Lentivirus-transfected SiHa and HeLa cells were seeded on coverslips overnight at 2 × 10^5^ cells/well in 6-well plates. Then, the cells were treated with 10 µM 2X EdU solution (Click-IT EdU imaging kit, Invitrogen, Carlsbad, CA, USA) and further incubated for 2 h. After incubation, the cells were fixed in 4% formaldehyde at room temperature for 15 min and then permeabilized with 0.25% Triton X-100 in PBS for 15 min. The cells were washed twice with 3% bovine serum albumin (BSA) in PBS and further incubated with Click-iT reaction cocktail containing Alexa Fluor azide 555 for 30 min at room temperature away from light. This was followed by washing with 3% BSA-PBS. Nuclear staining was carried out by incubating the cells with 5 µg/mL Hoechst 33342 for 30 min at room temperature, protected from light. EdU staining was analyzed by using the Zeiss LSM 880 Airyscan microscope at ×63 magnification. Image analysis was performed by using ImageJ v 1.54f software.

### XTT assay

The XTT assay was employed to evaluate the cell viability of lentivirus-transfected cells stably expressing shRNA targeting RB1 or control shRNA. The cells were seeded at 2,000 cells/100 µL DMEM/well into a 96-well plate and incubated for 48 h at 37°C. Then, 50 µL of the XTT labeling mixture (Cell Proliferation Kit II [XTT], Roche, Germany) was added to each well and further incubated for 5 h. Finally, the absorbance was read at 450 and 630 nm, and the absolute absorbance (OD_total_) was generated as OD_450_ – OD_630_. Then, the average value of OD_total_ collected from six wells per sample was calculated.

To compare the relative viability, all the data were presented as the mean percentage ± standard deviation (SD) of six replicates compared with the value of control cells. The cell viability was calculated as follows: cell viability (%) = (AOD_total_ shRNA RB1/AOD_total_ shRNA GFP) × 100, where AOD_total_ is the average value of the absolute absorbance as described above.

### Colony formation assay

Lenti-transfected SiHa, CaSki, HeLa, and HaCaT cells were seeded at 400 cells/plate into 6 cm^2^ plates. The cells were incubated for 15 d with media changes every 2 d. Then, colonies were fixed with 4% paraformaldehyde and stained with 10% Giemsa stain. After staining, photographs of the plates were taken, and colonies were counted using CellCount software ImageJ v 1.54f. The experiment was performed in triplicate and in two independent assays.

### Cell transfection for pRB restoration

SiHa and HeLa cells stably expressing shRNA targeting RB1 were seeded into 6-well plates at a density of 1.2 × 10^5^ cells/well. After 24 h, the cells were transfected with 1 µg of empty vector, wild-type pRB, or any of the three pRB mutants described above using a ratio of 1 µg of DNA:2 µL of FuGENE HD transfection reagent (Promega) according to the manufacturer’s instructions. All cells were co-transfected with LacZ to compare transfection efficiency. After further incubation for 48 h, the cell lysates were harvested and analyzed by Western blotting, and β-Gal was used as a loading control.

### Statistical analysis

Statistical analysis was performed using GraphPad Prism 5 Software. Functional comparisons between cells transfected with control siRNA/shRNAs and cells expressing siRNA/shRNAs targeting RB1 were performed using Student’s *t*-test. In all analyses, the differences were considered statistically significant whenever *P* < 0.05. All experiments were performed at least thrice, and data are shown as the mean ± standard deviation of the mean. *P* values are defined as follows: *P < 0.05, **P < 0.005, ***P < 0.001, “ns” represents a non-significant *P* value above 0.05.

The quantification of protein levels from Western blots was achieved by measuring band intensities using ImageJ v 1.54f software. The relative quantification values are the ratio of the net band of the target proteins to the net bands of the respective loading controls.

## RESULTS

### Knockdown of pRB destabilizes HPV E7 oncoprotein in cervical cancer cell lines

**Fig 1:**
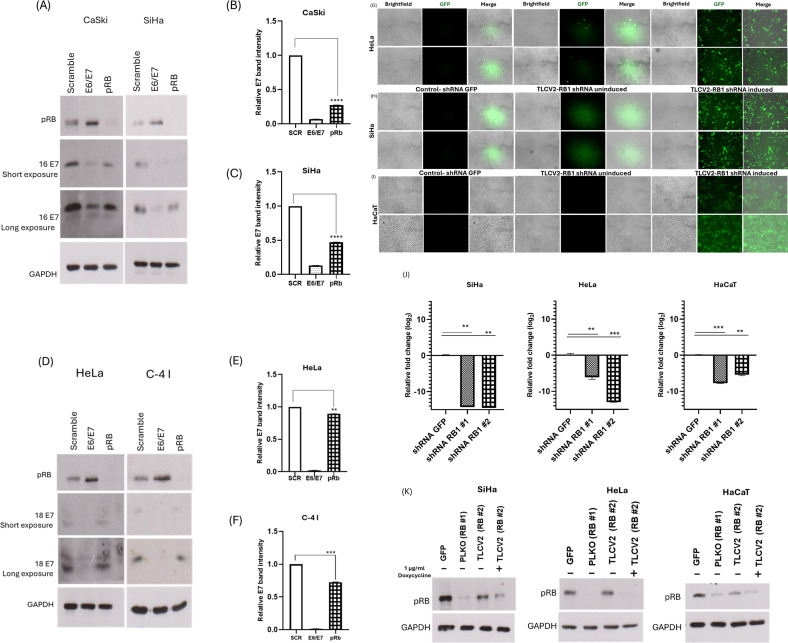
The transient knockdown of pRB significantly destabilizes HPV-16 and -18 E7. HeLa, CaSki, C-4 I, and SiHa cells, expressing endogenous HPV-16 or -18 E7 oncoprotein, were transfected with siRNAs targeting 18 E6/E7 (for HeLa and C-4 I), 16 E6/E7 (for CaSki and SiHa), and pRB. Scramble siRNA (SCR) was used as a control. The cells were harvested after 72 h, then pRB and E7 proteins were assessed by Western blotting (A and D) using antibodies specific to HPV-16 E7, HPV-18 E7, and pRB. GAPDH expression is shown as a loading control. The E7 band density was normalized to the GAPDH band density, and the data were used to plot the densitometry graphs (B, C, E, and F). Data are shown as the fold changes of normalized E7 levels relative to the control (SCR) ± standard error of the mean. *P* values of three independent experiments were calculated by Student’s *t*-test. (G–I) The transfection and expression efficiencies of the inducible GFP-tagged pRB shRNA (RB1 #2) in cervical cancer cell lines and normal keratinocytes were evaluated by observing the fluorescence of GFP. Representative micrographs of SiHa, HeLa, and HaCaT cells expressing control GFP shRNA and doxycycline-inducible Cas9-2A-eGFP-sgRNA RB1 are presented. Magnification: ×200. The contrast and brightness levels of the brightfield images were enhanced for better visualization using ImageJ. (J) pRB mRNA expression in the lentivirus-transfected cell lines was analyzed by RT-qPCR. Graphs show mRNA levels normalized to the control shRNA. (K) The expression of pRB protein in the lentivirus-transfected cell lines was analyzed by Western blot. *P < 0.05, **P < 0.005, ***P < 0.0005, ****P < 0.0001.

The role played by the E7-facilitated degradation of pRB in the proliferation of cancer cells has been extensively studied. This interaction results in pRB dysregulation, a common feature of cervical cancer (38). During our investigations on the selective interaction of HPV-16 E7 with pRB and AP2M1 (data not shown), we surprisingly observed that siRNA-mediated depletion of pRB resulted in lower levels of E7 oncoprotein in CaSki and SiHa cells (Fig. 1A through C). We also determined a similar effect on HPV-18 E7 in HeLa and C-4 I cells, albeit to a lesser extent (Fig. 1D through F).

To further investigate a possible pRB-mediated E7 stabilization in cervical-cancer-derived cell lines, we generated stable cell lines of SiHa, CaSki, HeLa, and C-4 I expressing control shRNAs or shRNAs targeting pRB (RB1) by lentiviral transduction and puromycin selection. All the cell lines had transduction efficiency above 85% (Fig. 1G through I), at least 60% pRB mRNA depletion (Fig. 1J) and at least 85% decrease in the protein levels of pRB with both shRNAs (Fig. 1K), as shown by fluorescence microscopy, RT-qPCR, and Western blotting. After 48 h of incubation, cells were harvested, and the whole cell lysates were analyzed by Western blotting.

**Fig 2:**
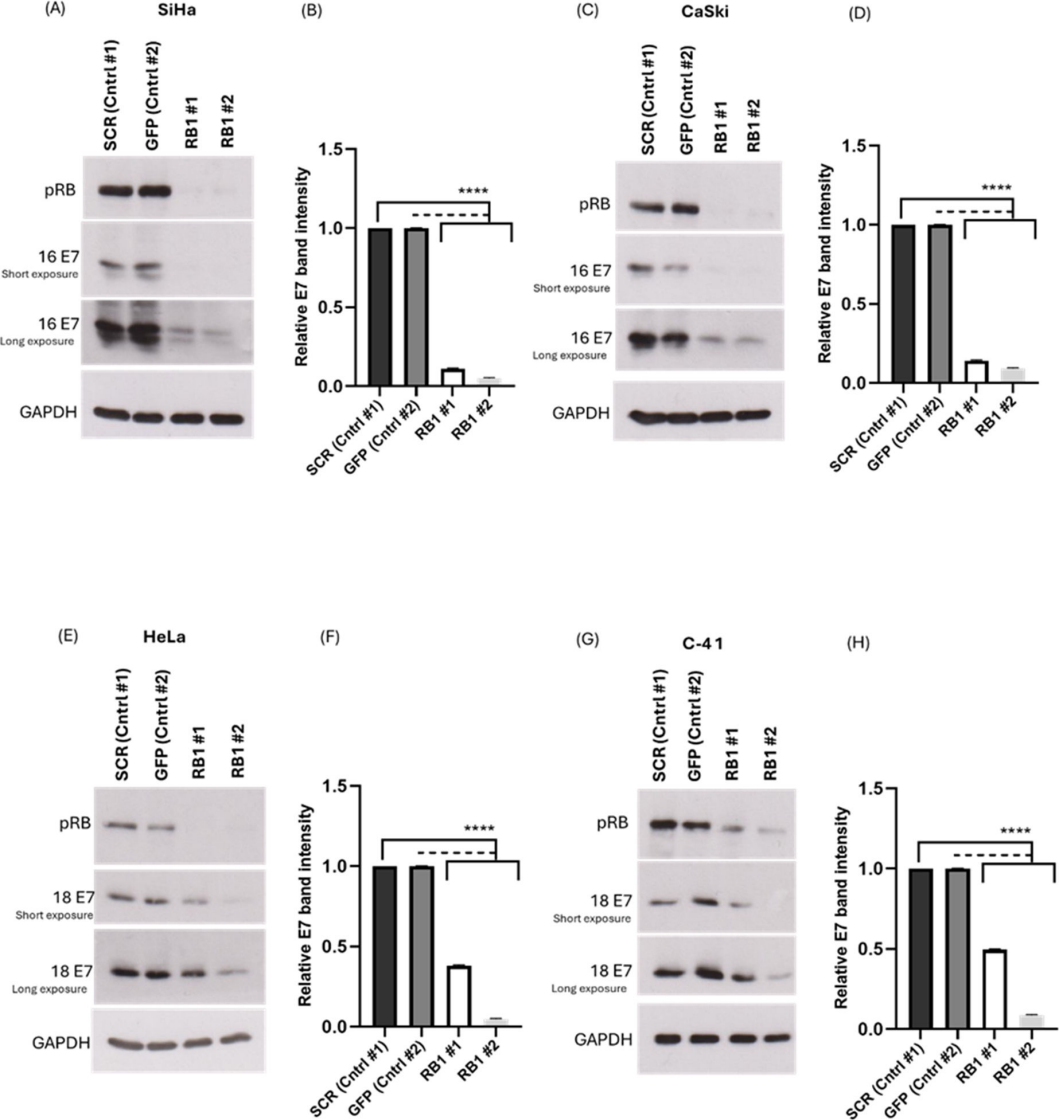
The knockdown of pRB significantly destabilizes HPV-16 and -18 E7 in stable shRNA RB1-transduced cells. (A and C) Endogenous HPV-16 E7 oncoprotein levels in transduced SiHa and CaSki cells were assessed by Western blotting using antibody specific to HPV-16 E7. (E and G) Endogenous HPV-18 E7 oncoprotein levels in transduced HeLa and C-4 I cells were assessed by Western blotting using antibody specific to HPV-18 E7. GAPDH expression is shown as a loading control. The E7 band density was normalized to the GAPDH band density, and the data were used to plot the densitometry graphs (B, D, F, and H). Data are shown as the fold changes of normalized E7 levels relative to the controls ± standard deviation. *P* values of three independent experiments were calculated by Student’s *t*-test. **P < 0.005, ***P < 0.0005, ****P < 0.0001.

As shown in Fig. 2, cells with a stable knockdown of pRB show significant diminution in the protein levels of both HPV-16 and HPV-18 E7, compared with the controls, similar to what we observed in the transient assays. A seemingly more pronounced decrease is seen in CaSki and SiHa cells, compared with C-4 I and HeLa cells. While the reasons for this are currently unclear, it could be related to differences in the affinity of association between the different E7 oncoproteins, pRB, and components of the cellular degradation machinery (39–41). Nevertheless, the transient knockdown and stable knockdown of pRB show similar phenotypes in cervical-cancer-derived cell lines, indicating that the effects are strongly associated with pRB depletion. Taken together, these results suggest that the presence of pRB is important for maintaining the levels of E7 protein in cervical-cancer-derived cell lines.

### pRB knockdown enhances proteasomal degradation of E7 oncoprotein

**Fig 3:**
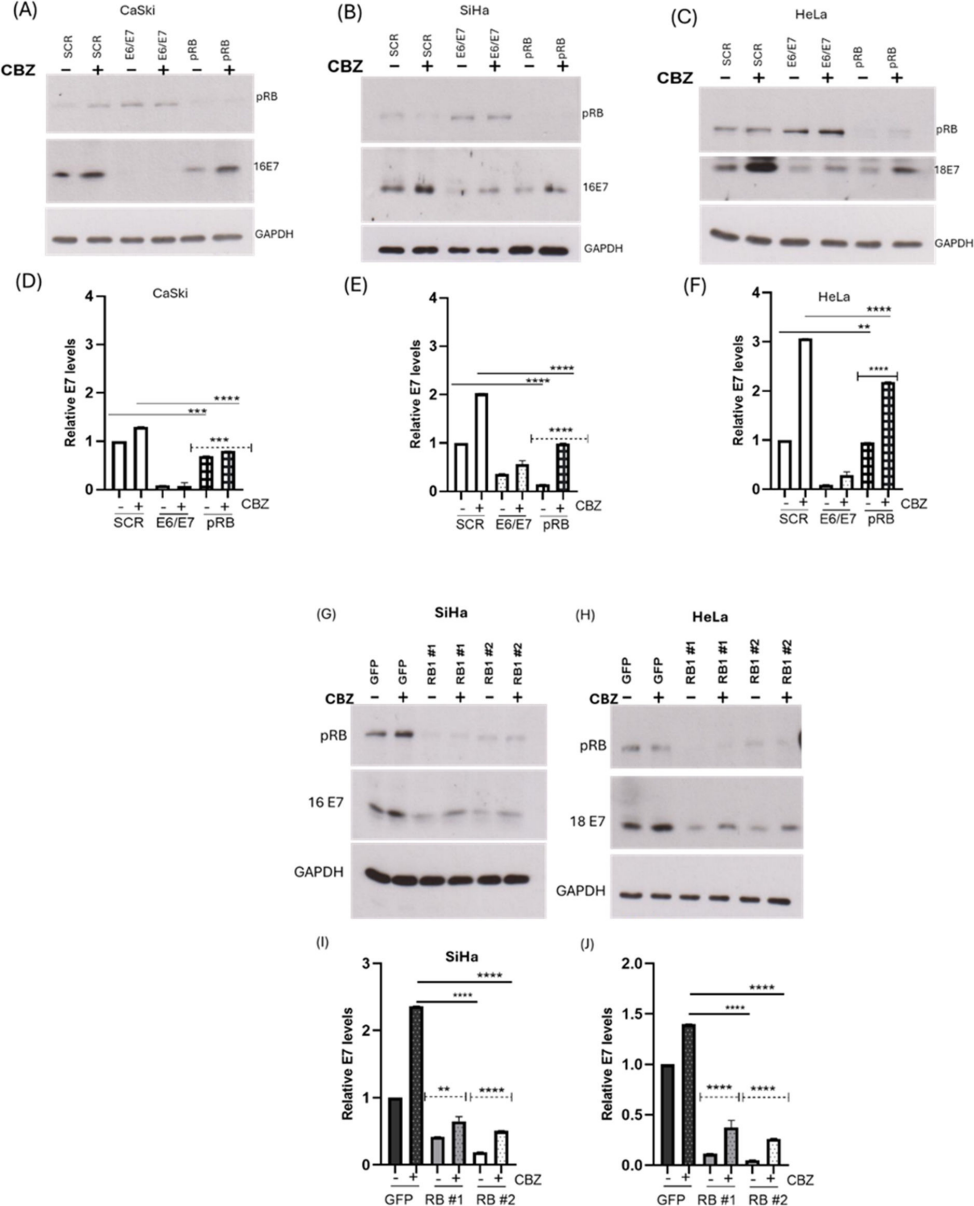
Proteasomal degradation is not compromised by pRB knockdown. (A–C) CaSki, SiHa, and HeLa cells were transfected with siRNAs targeting HPV-16/18 E6/E7 and pRB; Scramble siRNA (SCR) was used as a control. Cells were treated with DMSO (control) or proteasome inhibitor CBZ (20 µM) for 6 h after 72 h of siRNA transfection. (G–J) Transduced SiHa and HeLa cells were treated with DMSO or CBZ (20 µM) for 6 h. pRB and E7 levels were assessed by Western blotting using antibodies specific to HPV-16/18 E7 and pRB (G and H). GAPDH expression is shown as a loading control. The E7 band density was normalized to the GAPDH band density, and the data were used to plot the densitometry graph (D–F, I, and J). Data are shown as the fold changes of normalized E7 levels relative to the control (GFP) ± standard deviation. *P* values of three independent experiments were calculated by Student’s *t*-test. **P < 0.005, ***P < 0.0005, ****P < 0.0001.

To investigate the mechanism of E7 destabilization caused by pRB knockdown, we assessed the effect of inhibiting proteasomal function on HPV-16 and HPV-18 E7 levels using transiently and stably transfected cells. For the transient assay, after 72 h of siRNA transfection to knock down pRB in CaSki, SiHa, and HeLa cells, the cells were treated with the proteasome inhibitor CBZ, or dimethyl sulfoxide (DMSO) as a control, followed by a further incubation of 6 h. The cells were then harvested, and E7 protein levels were analyzed using Western blotting. We found that blocking proteasome function results in the notable accumulation of E7 levels in cells transfected with the control and pRB siRNAs (Fig. 3A through F), although rescue never reached the levels seen in the cells treated with control siRNA.

To further validate this observation, transduced SiHa and HeLa cells were treated with CBZ or DMSO and were analyzed as described above. As shown in Fig. 3G through J, the transduced cells also showed accumulation of E7, but like the transient assay, the accumulated E7 levels in the pRB-depleted cells were lesser than in the control cells. In both transient and stable knockdown cells, the addition of the proteasomal inhibitor did not restore E7 levels to those in pRB-positive cells as would be expected if turnover is solely via the proteasome. Hence, the mechanism of pRB-mediated E7 destabilization is not entirely due to enhanced proteasomal degradation.

### pRB knockdown selectively downregulates E7 transcription

**Fig 4:**
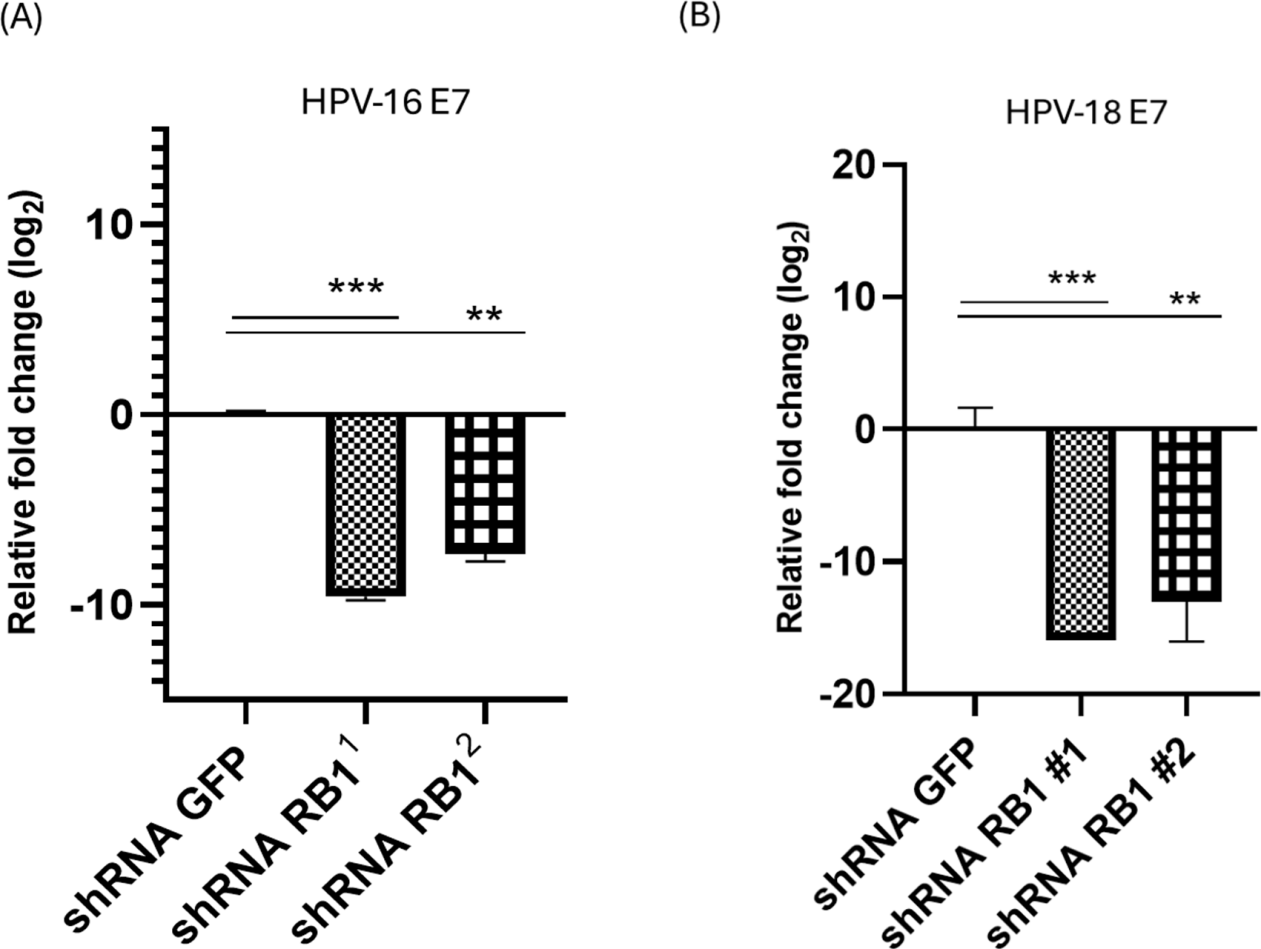
E7 mRNA levels are perturbed by pRB knockdown. SiHa (A) and HeLa (B) cells expressing control shRNA or shRNA targeting RB1 were grown in 10 cm^2^ plates. After 48 h, the cells were harvested, and total RNA was extracted and reverse transcribed to generate cDNA, then used in a real-time PCR amplification of E7 transcripts. Graphs show mean fold changes of E7 mRNA levels in the presence and absence of pRB. GAPDH was used as an internal control in the relative quantification of the genes. *P* values of three independent experiments were calculated by Student’s *t*-test. **P < 0.005, ***P < 0.0005.

Due to the failure of proteasomal inhibition to completely restore E7 levels in the absence of pRB, we decided to check mRNA levels of E7 in the transduced cells with and without pRB depletion. We found that there was significant downregulation of E7 mRNA levels in both SiHa (Fig. 4A) and HeLa (Fig. 4B) cells. This suggests that the knockdown of pRB seems to perturb the transcription of E7. It is, however, unclear yet whether this occurs as a direct effect or an indirect one through other transcriptional factors such as AP-1 and Brg-1, which have been shown to modulate the expression of E7 in HPV-infected cells (42, 43).

### pRB knockdown significantly reduces the half-life of HPV-16 and -18 E7

**Fig 5:**
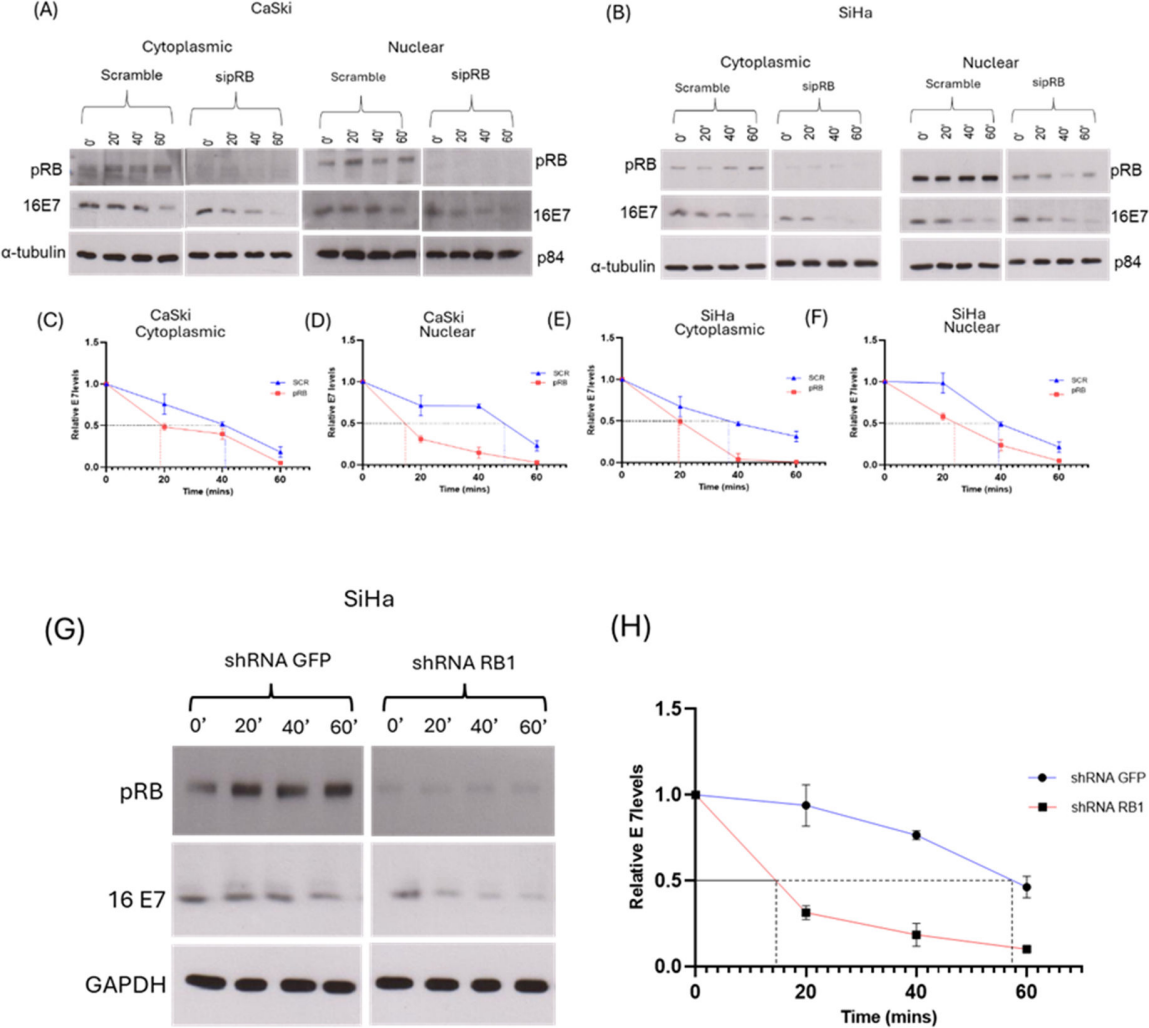
pRB depletion reduces the half-life of HPV-16 E7. (A and B) CaSki and SiHa cells were transfected with sipRB, and Scramble siRNA (SCR) was used as a control. After 72 h, the cells were treated with 50 µg/mL CHX for 0, 20, 40, or 60 min. The cells were then harvested at the different timepoints and processed into cytoplasmic and nuclear fractions. pRB and E7 levels were assessed by Western blotting using antibodies specific to HPV-16 E7 and pRB. α-Tubulin and p84 were used as cytoplasmic and nuclear loading controls, respectively. The E7 band density was normalized to the tubulin or p84 band density, and the data were used to plot the densitometry graphs (C–F). (G) SiHa cells transduced with shRNA targeting pRB or shRNA GFP as a control were treated with CHX as described. GAPDH was used as a loading control. The E7 band density was normalized to the GAPDH band density, and the data were used to plot the densitometry graph (H). Data are shown as the fold changes of normalized E7 levels, relative to the control (GFP) ± standard deviation. Dashed lines represent the half-life of E7 in the absence of pRB while dotted lines represent the half-life of E7 in the control

Having shown that pRB knockdown downregulates both E7 protein and mRNA, we next asked how much of the lower levels of E7 protein is due to increased E7 protein turnover. To do this, we assessed the posttranslational stability of the E7 protein by performing cycloheximide chase (half-life) assays in CaSki and SiHa cells in the presence and absence of pRB, using siRNA against pRB and Scramble siRNA as a control. We also wanted to determine the trend of turnover in the cytoplasmic and nuclear pools of E7 in the cells. After 72 h, the cells were treated with cycloheximide to prevent *de novo* protein synthesis. The cells were then harvested at 0, 20, 40, and 60 min posttreatment, followed by cell fractionation. The cytoplasmic and nuclear fractions were analyzed by Western blotting for HPV-16 E7 levels. As seen from Fig. 5A through F, the half-life of HPV-16 E7 in CaSki and SiHa cells transfected with Scramble siRNA lies between 36 and 60 min in both cytoplasmic and nuclear pools, as previously reported (44, 45). However, in cells transfected with siRNA targeting pRB, HPV-16 E7 half-life is reduced twofold and lies between 18 and 20 min in the cytoplasmic fractions, and between 14 and 20 min in the nuclear fractions.

The assay was also performed using transduced SiHa cells in the presence and absence of pRB, using shRNA against pRB and shRNA GFP as a control. After 24 h of incubation, the cells were treated with cycloheximide and processed as described above but without fractionation. As seen from Fig. 5G and H, the half-life of HPV-16 E7 in cells expressing control shRNA lies between 56 and 60 min while E7 half-life in cells expressing shRNA targeting pRB lies between 14 and 16 min. Taken together, these results indicate that the knockdown of pRB expression causes accelerated turnover of E7, and the cytoplasmic and nuclear pools of E7 are similarly impacted. This suggests that the major explanation for the decreased E7 levels in the cervical cancer cell lines upon pRB ablation is most likely due to protein destabilization rather than a consequence of decreased levels of transcripts.

### pRB knockdown inhibits cell growth

**Fig 6:**
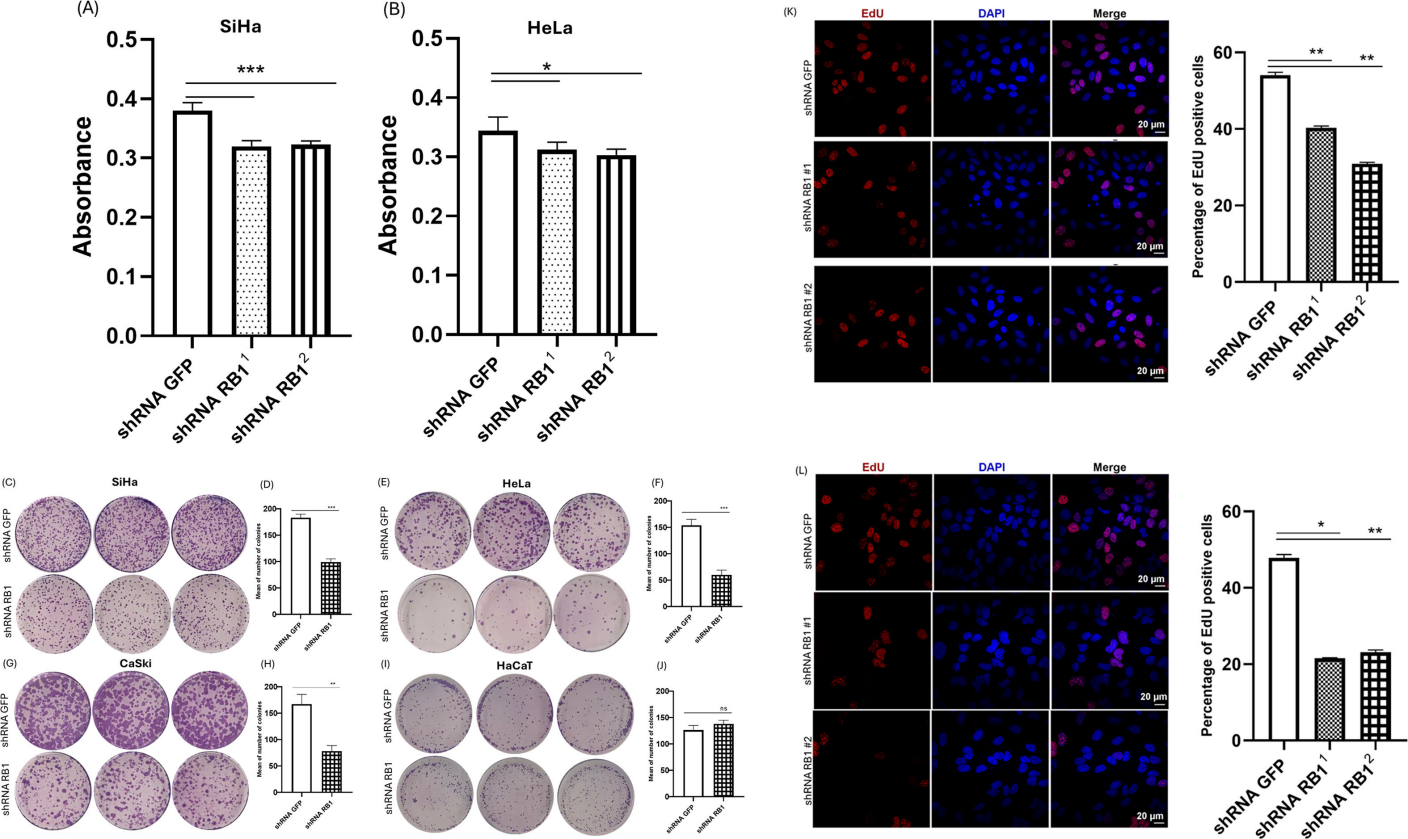
The knockdown of pRB caused a marked decrease in the proliferation of SiHa and HeLa cells. (A and B) SiHa and HeLa cells transduced with shRNAs targeting pRB or GFP shRNA as a control, seeded in equal numbers, were subjected to XTT assay after 48 h of incubation. (C–J) SiHa, CaSki, HeLa, and HaCaT cells were seeded in equal numbers in 6 cm^2^ plates. Cells were further incubated for 15 d, followed by staining with 10% Giemsa stain. Each experiment was performed in triplicate and in at least two independent assays. Photographs of plates from colony formation assay by direct plating and the quantification of the relative number of colonies obtained from the average counts of colonies from three plates per shRNA. The quantification graphs are presented as fold changes in colony formation relative to the control (GFP) ± standard deviation or means of colonies ± standard deviation. (K and L) Micrographs and quantification of EdU-labeled SiHa and HeLa cells. *P* values of three independent experiments were calculated by Student’s *t*-test. **P < 0.005; ***P < 0.0005; ns, not significant.

To further explore the effect of pRB knockdown on cervical cancer cell lines, we evaluated the proliferation of the cells in the presence or absence of pRB. E7 oncoprotein promotes cell proliferation, and studies have shown that the siRNA-mediated knockdown of E7 in cancer cell lines induces cell growth arrest. Using XTT, the effects of pRB ablation were detected in transduced SiHa and HeLa cells. As shown in Fig. 6A and B, the XTT assay revealed that the knockdown of pRB markedly decreased SiHa and HeLa cell growth.

To further validate the effect of E7 destabilization on cell proliferation, we hypothesized that the decrease in E7 levels caused by pRB knockdown would decrease clonogenicity if, indeed, E7 is regulatorily perturbed. To address this, we assessed the *in vitro* cellular transformation of pRB-knockdown cells by performing colony formation assay in anchorage-dependent conditions. Transduced SiHa, CaSki, HeLa, and HaCaT cells stably expressing pRB shRNA or control shRNA were seeded in equal numbers of cells and incubated for 15 d. As shown in Fig. 6C through J, pRB knockdown caused a marked decrease in the number and size of colonies formed in SiHa, CaSki, and HeLa cells but not in the non-cancerous HaCaT cells (Fig. 6F), which is probably due to decreased levels of E7 in the HPV-positive cells.

Additionally, we performed EdU labeling assay on transduced SiHa and HeLa cells. Indeed, EdU-positive cells were significantly lesser in cells with pRB knockdown, as shown in Fig. 6K and L. These findings, therefore, suggest that the continued expression of pRB is crucial for the proliferation of cervical cancer cells and cellular transformation mediated by E7.

### Overexpression of pRB restores E7 levels in SiHa and HeLa cells

**Fig 7:**
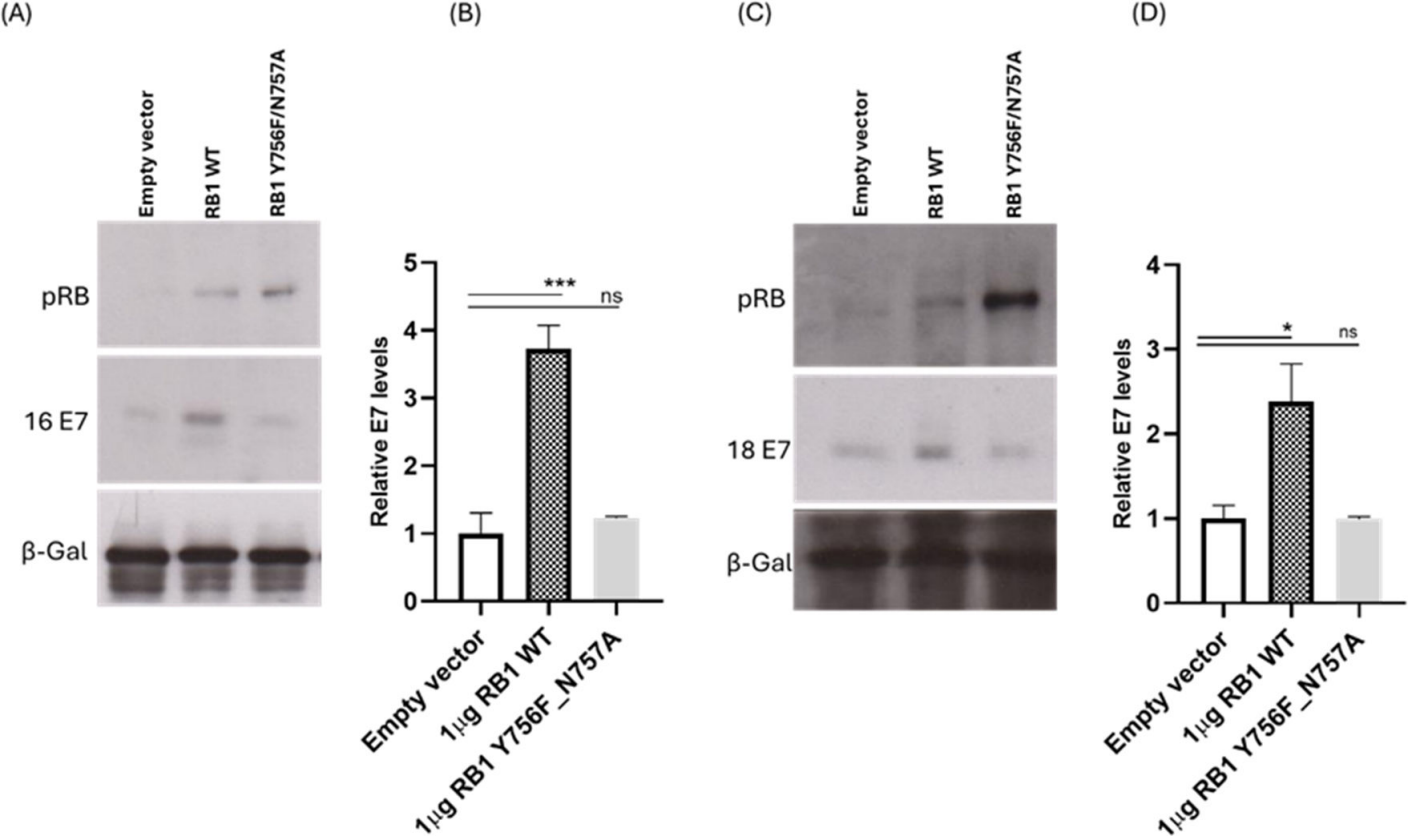
Overexpression of wild-type pRB restores E7 levels in PRB-knockdown cells. SiHa (A) and HeLa (C) cells expressing shRNA targeting RB1 were transfected with wild-type pRB or mutant pRB. After 48 h, cells were harvested and assessed by Western blotting. Beta galactosidase (β-Gal) expression is shown as a loading control. The E7 band density was normalized to the β-Gal band density, and the data were used to plot the densitometry graphs (B and D). Data are shown as the fold changes of normalized E7 levels relative to the controls ± standard deviation. *P* values of three independent experiments were calculated by Student’s *t*-test. **P < 0.005; ***P < 0.0005; ****P < 0.0001; ns, not significant.

Having shown that the knockdown of pRB perturbs E7 protein levels, we then investigated whether restoring pRB to residual levels as seen in wild-type cervical cancer cell lines would impact E7 levels. To do this, we transfected SiHa and HeLa cells with a stable knockdown of pRB, with wild-type pRB. Interestingly, we observed a significant restoration of E7 levels in both SiHa and HeLa cells as shown in Fig. 7. As an additional control, the pRB-knockdown cells were transfected with a pRB mutant with disruptions at the LXCXE binding cleft, RB1 Y756F_N757A, which does not bind E7 (34, 35). The Y756F_N757A mutant correspondingly fails to restore E7 levels. These results indicate that the pRB regulation of E7 stability is mediated in part by the direct protein–protein interaction.

## DISCUSSION

The E7 oncoprotein is essential for HPV replication (3), and its sustained expression at relatively high levels contributes to carcinogenic transformation in HPV-associated cancer progression (26, 46). One of the mechanisms by which HR HPV, specifically HPV-16 and HPV-18 E7, facilitates carcinogenesis is the degradation of the tumor suppressor, pRB. Previous studies have shown that the extent of pRB degradation is dependent on E7 protein levels, and that increased E7 expression can antagonize pRB functionality in cervical cancer cell lines (26, 46). While pRB is a known degradation target of HR HPV to repress the cell cycle suppressive capacity of the host, we report a new regulatory role of pRB—one that resembles a chaperone activity, whereby pRB contributes toward the stabilization of HPV-16 and -18 E7 oncoproteins.

We found that silencing pRB through RNA interference results in a significant decrease in the levels of HPV-16 E7 and HPV-18 E7, although the destabilization of E7 is more marked with HPV-16 than with HPV-18. The reasons for this difference between HPV-16 and HPV-18 E7 remain unclear but could be a reflection of differences in affinity for pRB between HPV-16 E7 and HPV-18 E7 with pRB, as well as potential differences in how the two oncoproteins interact with the cellular degradation machinery (40, 41).

It is noteworthy that while proteasome protection rescued a substantial amount of E7, the rescued levels never attained control levels. In order to investigate other potential explanations for the loss of E7, we found that cells deficient in pRB show reduced mRNA levels for E7. Previous studies have shown that E7 mRNA levels can be more sensitive to changes in cell cycle regulation or transcription factors such as AP-1 (42, 47–50). pRB binds to members of the AP-1 family of transcription factors, including c-Jun, and stimulates c-Jun transcriptional activity (51, 52). Similarly, HPV-16 E7 protein binds to c-Jun and inhibits the ability of pRB to activate c-Jun (51, 53). These interactions may be relevant for E7 mRNA modulation in response to the loss of pRB. The regulation of E7 by pRB clearly includes both transcriptional and posttranslational effects. Using cycloheximide chase assays, we showed a 50% decrease in the half-life of HPV-16 E7 in SiHa cells when pRB expression is silenced. Furthermore, this increased turnover appears to be proteasome mediated.

This study also showed that the knockdown of pRB significantly repressed cell proliferation in cervical cancer cell lines. This was surprising because in the context of cervical cancer, where HPV E7 oncoprotein already inactivates pRB, it could be posited that the knockdown of pRB would further exacerbate the disruption of normal cell cycle checkpoints, contributing to uncontrolled proliferation. Contrastingly, DNA synthesis, cell proliferation, and colony formation of SiHa and HeLa cells declined upon the knockdown of pRB, which we believe may be attributable to the destabilization of E7. Moreover, previous studies have shown that the downregulation of E7 oncoprotein inhibits carcinogenesis by arresting cell cycle progression and proliferation, and inducing anoikis in HPV-positive cancer cell lines (54, 55). Together, these results show that the destabilization of E7 due to pRB loss is sufficient to reduce its clonogenic potential. Thus, indicating that the homeostatic level of pRB is essential for the survival of cervical cancer cells. The therapeutic implications of this regulatory role of pRB on E7 thus require further investigation.

Having established that the continued expression of pRB and the maintenance of its residual protein levels in HPV-positive cervical cancer cell lines is important for E7 stabilization and cell viability, we sought to investigate how much of the effect was due to a direct protein interaction between pRB and E7. Cells were transfected with wild-type pRB and Y756F_N757A mutant, which destroys interaction with E7 (34, 35, 56, 57). Intriguingly, the restoration of wild-type pRB in the knockdown cells restored E7 levels in both SiHa and HeLa cells stably expressing shRNA against pRB; however, the non-E7-binding pRB mutant failed to restore E7. These results indicate that the loss of pRB interaction with E7 is responsible in part for the reduction in E7 levels and its enhanced degradation at the proteasome.

Overall, this study provides new insights into the E7–pRB interaction in cervical cancer cells and indicates that pRB has a regulatory role in controlling E7 levels, in a manner that is mediated at both the transcriptional and posttranslational levels.

## Data Availability

All data supporting the findings of this study are included within the article or are available from the authors upon reasonable request.
